# Serotonin syndrome caused by a CYP2C19-mediated interaction between low-dose escitalopram and clopidogrel: a case report

**DOI:** 10.3389/fpsyt.2023.1257984

**Published:** 2023-10-10

**Authors:** Jianhong Wu, Jiao Yu, Kankan Qu, Jiajun Yin, Chunming Zhu, Xiaowei Liu

**Affiliations:** ^1^The Affiliated Mental Health Center, Jiangnan University, Wuxi, China; ^2^Wuxi Central Rehabilitation Hospital, Wuxi, Jiangsu, China

**Keywords:** serotonin syndrome, CYP2C19, escitalopram, clopidogrel, case report

## Abstract

**Background:**

Serotonin syndrome has been recognized as a serious adverse reaction to antidepressants and is characterized by sudden or severe autonomic nerve dysfunction and neuromuscular symptoms. Without an accurate diagnosis and prompt treatment, serotonin syndrome progresses rapidly and can be life-threatening. It is usually related to the dose of 5-hydroxytryptamine drugs, and the dose is the basis for diagnosis. Therefore, serotonin syndrome induced by low-dose antidepressants rarely occurs, and clinicians are more likely to misdiagnose patients who take low-dose antidepressants with similar symptoms. Here, we present a case study of serotonin syndrome caused by a relatively low dose of escitalopram, which is not common in past references.

**Case summary:**

The patient was a 74-year-old Asian woman with a 42-year history of schizophrenia. After 6 weeks of antidepressant treatment, our patient presented with characteristic myoclonus in the lower limbs and closed eyes with fluttering. Initially, she was misdiagnosed with neuroleptic malignant syndrome (NMS) due to antipsychotic medication and was treated accordingly, even with discontinuation of clozapine. However, her symptoms persisted, and then therapeutic drug monitoring was initiated with the involvement of a clinical pharmacist. Eventually, she was diagnosed with serotonin syndrome due to escitalopram levels reaching the warning level. Subsequently, the patient’s treatment was modified, and her clinical outcome was satisfactory without any other serious adverse reactions. Gene detection was also performed, and a cytochrome P450 enzyme (CYP) 2C19-mediated interaction between low-dose escitalopram and clopidogrel seems to be a possible mechanism.

**Conclusion:**

Data on this is extremely scarce, and to the best of our knowledge, serotonin syndrome caused by low-dose antidepressants has not yet been discussed to any great extent in the literature. Our case provides more clinical experience in the treatment of serotonin syndrome.

## Introduction

Serotonin syndrome is a potentially life-threatening adverse drug reaction caused by excessive 5-hydroxytryptamine (5-HT) activity in the synaptic space, which results in a variety of symptoms involving the central and peripheral nervous systems (1). Changes in mental state, autonomic nerve dysfunction, and neuromuscular symptoms such as myoclonus, ocular flutter, myotonia, and hyperreflexia are some of its typical manifestations (2). Its symptoms are diverse and easily confused, making diagnosis and treatment difficult.

The formation of 5-HT is the most important link in the pathogenesis of serotonin syndrome. 5-HT is produced by the hydroxylation and decarboxylation of L-tryptophan (3). Its amount and function are tightly regulated by reuptake inhibition mechanisms, feedback loops, and metabolic enzyme combinations. Initially, 5-HT is stored in vesicles by vesicular monoamine transporters after generation. When nerve cells are stimulated by the outer axon, 5-HT is released into the synaptic space. At the same time, the presynaptic 5-HT receptor acts as a feedback loop to inhibit the exocytosis of vesicles (4). The released 5-HT binds to the prominent posterior membrane receptors and produces a corresponding effect. Meanwhile, a reuptake inhibition mechanism returns 5-HT to the cytoplasm of presynaptic neurons, where it is reabsorbed by vesicles and then metabolized by type A monoamine oxidase (5). When the reuptake is inhibited, the level of 5-HT in the synaptic cleft increases (6). Excessive 5-HT activation binds to the appropriate receptors when present in doses above a threshold, leading to serotonin syndrome and a range of symptoms. As different 5-HT receptors are distributed on different types of neurons, such as the cerebral cortex, hypothalamus, gastrointestinal tract, blood vessels, and bronchial smooth muscle (7, 8), the symptoms of serotonin syndrome tend to be diverse.

However, the variety of symptoms makes diagnosis difficult, and many conditions have similar symptoms to those of serotonin syndrome. Without careful identification, it is easy to misdiagnose, especially in patients with mental disorders who are taking multiple antipsychotic drugs at the same time. Neuroleptic malignant syndrome (NMS) is an idiosyncratic reaction to a dopamine antagonist that is often misdiagnosed as serotonin syndrome (2). Due to their different treatment methods and withdrawal strategies, clinical misdiagnosis can lead to serious outcomes. In addition, serotonin syndrome is often caused by high-dose antidepressants or a drug combination containing higher levels of 5-hydroxytryptamine (9), and low-dose antidepressant-induced serotonin syndrome rarely occurs.

Here, we present a case study of serotonin syndrome caused by a relatively low dose of an antidepressant. Serotonin syndrome occurred despite medication, with escitalopram at only 5 mg/day. Because of the routine antipsychotic treatment, at first, the patient was mistakenly diagnosed with NMS. Eventually, therapeutic drug monitoring (TDM) and genetic testing helped us identify serotonin syndrome and explain the possible pathogenesis.

## Case description

### Patient presentation

The patient was a 74-year-old Asian woman with a 42-year history of schizophrenia. On February 23, 2023, she was admitted to the hospital due to auditory hallucinations and a behavioral disorder, which had been worsening for 2 days. Her medical history was as follows (Table 1).

**Table 1:** Medical history of our patient.

Time	Etiology	Presentation	Diagnosis	Treatment	Prognosis
December 1980	Acute mental disorders following postpartum infection with high fever	Insomnia, hooey, irritability, and aggressive behavior	Schizophrenia	Chlorpromazine, perphenazine (Dose is unclear)	Stable and able to resume work
October 1984	Self-induced medication withdrawal and relapse	Insomnia, talking to self, disorganized behavior and speech, aggressive behavior, and inability to care for herself	Schizophrenia	Chlorpromazine 600 mg qd, perphenazine 32 mg qd	Stable and able to resume work
Hospitalized three times (Exact dates are unknown)	Self-induced medication withdrawal and relapse	Insomnia, talking to self, hooey, aggressive behavior, and inability to care for herself	Schizophrenia	Chlorpromazine 450 mg qd	Stable
July 2007	Self-blame and guilt after fatigue	Insomnia, crying alone, socially withdrawn, staying away from family, inability to care for herself, and aggressive behavior	Schizophrenia with depression	Clozapine 300 mg qd	Stable, and can do housework, with medication adherence
October 24, 2015	Depressed about the death of her father	Poor mood, often self-blame	Schizophrenia with depression	Clozapine, amisulpride (Dose is unclear)	Stable
(The exact date is unknown)	The patient discontinued amisulpride after discharge and only took clozapine				
July 2022	Refuses medication	Socially withdrawn, disorganized behavior	Schizophrenia	Clozapine 250 mg qd, risperidone 3 mg qd	Stable
February 2023	Self-blaming and depressed because her husband needed to undergo surgery. She had suddenly banged her head against the wall after dinner.	Self-blame and guilt, melancholy, and self-harm	Schizophrenia	Clozapine 275 mg qd, risperidone 3 mg qd	

### Treatment course

After admission, the patient was treated with clozapine 275 mg/day, and risperidone 3 mg/day in combination with clopidogrel 75 mg/day because of her history of cerebral infarction. In addition, our patient was receiving rosuvastatin 10 mg qn and estazolam 1 mg qn for hyperlipidemia and insomnia, respectively. She had a history of hypertension and was not receiving antihypertensive medication because her current blood pressure was normal. However, 4 days after admission, her blood pressure was 188/95 mmHg and she was prescribed valsartan 80 mg/day. On the same day, our patient was less active and depressed, so risperidone was discontinued and escitalopram 5 mg/d was added to improve depression. During treatment, the patient received intermittent potassium chloride for her persistent hypokalemia.

After 6 weeks of admission (April 13, 2023), our patient incidentally showed insomnia, agitation, refusal to eat, nervous expression, tachypnea, tremor in the lower extremities, confusion, negativism, and diaphoresis, with neurological pathological signs being all negative. Vital signs were as follows: blood pressure 196/106 mmHg, heart rate 118 beats/min, respiratory rate 30 breaths/min, and temperature 36.7°C. Initial physical examination revealed: eyes closed with fluttering; increased muscle tension; myoclonus; hyperreflexia; and bilateral plantar tremor. Laboratory findings showed that creatine kinase (CK) was 267.2 IU/L. Combined with her symptoms and medications, she was initially diagnosed with neuroleptic malignant syndrome (NMS) (Table 2).

**Table 2:** Results of laboratory tests.

Variable	Value (reference range)
Blood chemistry and serology	
Total protein (g/L)	77.9 (60.0–83.0)
Albumin (g/L)	42.1 (35.0–50.0)
Globulin (g/L)	**35.8** (15.0–35.0)
Prealbumin (mg/L)	306.0 (200.0–400.0)
Total bilirubin (umol/L)	13.0 (0–23.0)
Direct bilirubin (umol/L)	3.2 (0–7.0)
Aspartate aminotransferase (IU/L)	21 (5–50)
Alanine aminotransferase (IU/L)	20 (7–40)
Alkaline phosphatase (U/L)	92.8 (40–150)
Lactate dehydrogenase (IU/L)	**264** (109–245)
γ-Glutamyltransferase (IU/L)	25 (7–50)
Sodium (mmol/L)	142.2 (137.0–147.0)
Potassium (mmol/L)	3.09 (3.5–5.3)
Chloride (mmol/L)	101.6 (99.0–110.0)
Creatinine (umol/L)	63.7 (45.0–104.0)
Urea nitrogen (mmol/dL)	7.2 (2.9–8.2)
Creatine kinase (IU/L)	**267.2** (26–174)
Creatine kinase-MB fraction (IU/L)	**25.1** (0–25.0)
Hematology	
Red blood cells (×10^12^/L)	4.85 (3.5–5.13)
Hemoglobin (g/L)	**151** (110–150)
Hematocrit (%)	45.1 (34.0–51.0)
White blood cells (×10^9^/L)	**19.7** (4.0–10.0)
Platelet count (×10^9^/L)	**348** (100–300)
Neutrophil percentage (%)	**92.6** (50–70)

The bold values means pathologic changes in the laboratory report.

**Figure 1:**
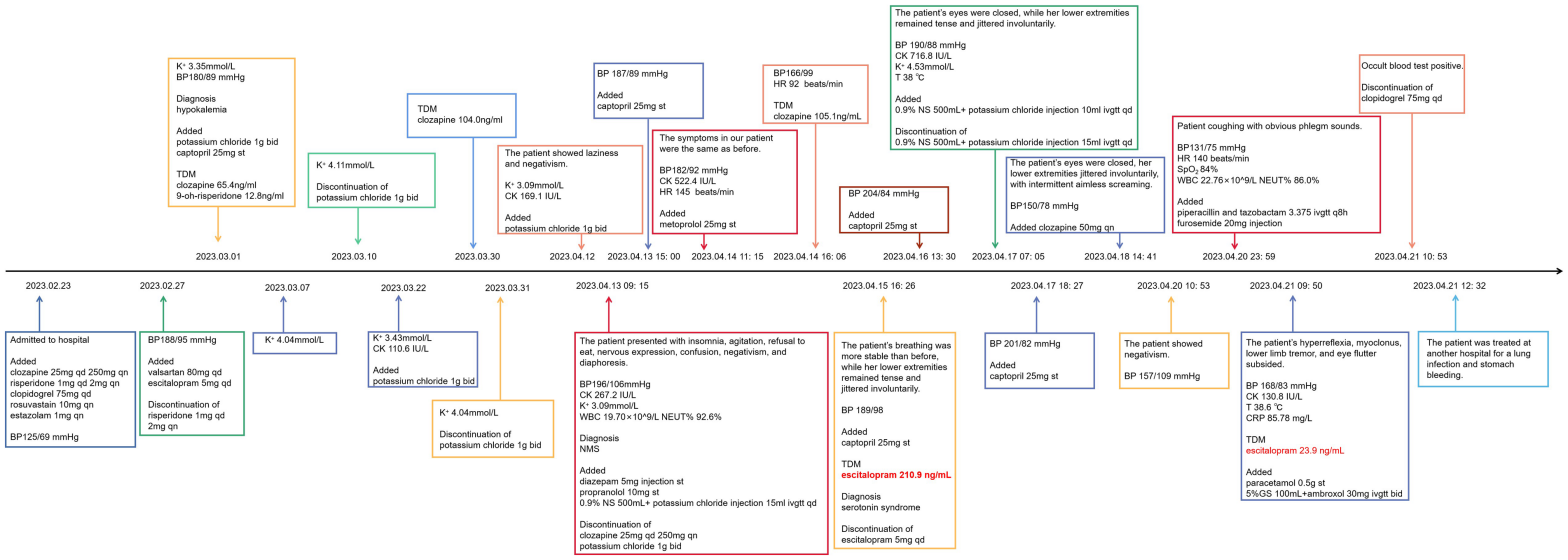
Case report timeline.

Intravenous fluids, diazepam 5 mg injection, and propranolol 10 mg were started to relieve the symptoms, and the clozapine was immediately stopped. However, there was little change in her symptoms over the following 24 h. Moreover, what confused us was that the concentration of clozapine in our patient was 105.1 ng/mL, much lower than the normal level. On April 15, 2023, although the patient’s breathing was more stable than before, her lower extremities remained tense and jittered involuntarily. The timeline for our patient was as follows (Figure 1).

Such a triad of autonomic dysfunction, neuromuscular excitement, and mental state changes could also be considered a serotonin syndrome, according to the literature (10). Therefore, TDM and gene detection were introduced despite the low dose of escitalopram. The results showed that the escitalopram level was 210.9 ng/mL, which was much higher than the cutoff, and the polymorphism of CYP2C19 was typical of poor metabolizers (PMs). Combined with the patient’s current symptoms, she was diagnosed with serotonin syndrome (Table 1).

**Figure 2:**
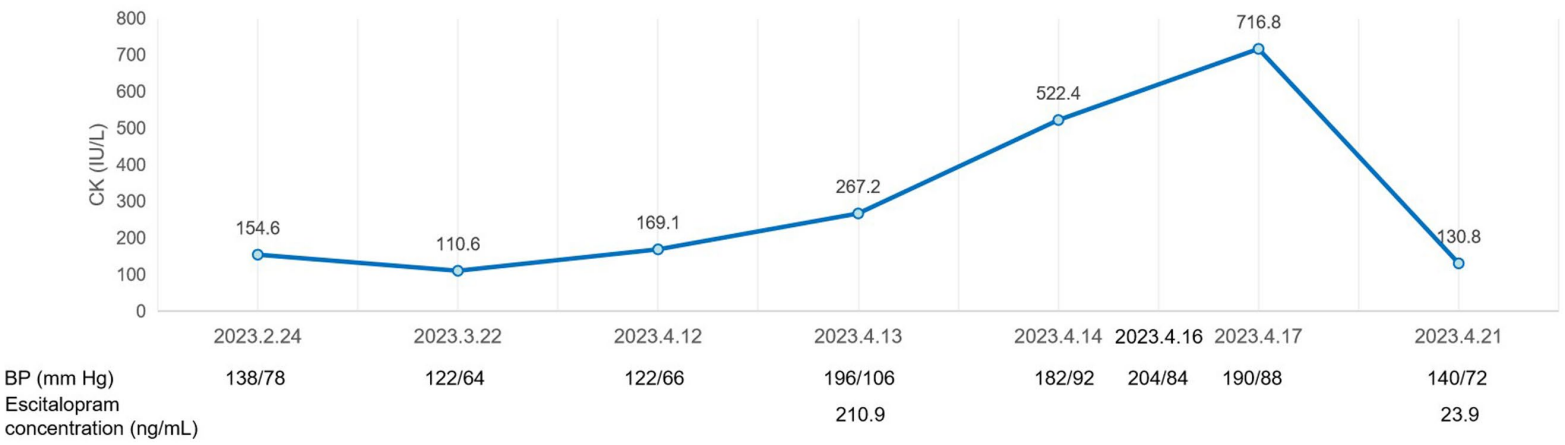
A graphic representation of the clinical course of the patient with serotonin syndrome, showing laboratory values during hospitalization.

Escitalopram was then discontinued. In the following 2 days, the patient’s blood pressure rose to 204/84 mmHg, and heart rate reached 145 beats/min. Intravenous fluids were used to promote drug metabolism, and captopril 25 mg was given orally as needed for antihypertensive therapy. The patient’s myoclonus persisted, and her CK increased to 716.8 IU/L on April 17. Given that the half-life of escitalopram is 30 h, its metabolites last longer, and an elderly patient needs more time to eliminate escitalopram, the increase in laboratory results 2 days after her discontinuation seemed to be normal (Figure 2). Over the next 4 days, the patient presented with intermittent jittering, negativism, and myoclonus. On April 21, 2023, her hyperreflexia, myoclonus, lower extremities tremor, and ocular flutter subsided, blood pressure gradually returned to 140/72 mmHg; CK returned to normal; and her escitalopram level decreased to 23.9 ng/mL. Subsequently, the patient developed other physical conditions, which included recurrent pneumonia and gastric bleeding, and was admitted to another hospital. After the completion of treatment, she returned to our hospital and was discharged in the following weeks.

## Discussion

At present, with the substantial increase of antidepressant drugs such as selective serotonin reuptake inhibitors (SSRIs), and 5-HT and NE reuptake inhibitors (SNRIs), the burden of serotonin in patients is increasing, and serotonin syndrome has become an urgent clinical problem (11). Due to its diverse and non-specific manifestations, serotonin syndrome is easily ignored, misdiagnosed, or exacerbated if not carefully evaluated, especially in elderly patients with a prescribing cascade. For example, hypertension caused by serotonin syndrome may be considered to be a deterioration of the patient’s primary condition, while tremor may be regarded as common adverse reaction. Anxiety and increased muscle tone may be mistakenly attributed to the patient’s mental state. Although the early clinical symptoms of serotonin syndrome are often mild to moderate, individuals can quickly deteriorate without active recognition and care (12). Therefore, it is necessary to improve clinicians’ understanding of serotonin syndrome.

According to the Hunter criteria, the typical clinical manifestation of serotonin syndrome is a triad of autonomic dysfunction, neuromuscular excitement, and mental state changes (13). Clonus (spontaneous, inducible, and ocular) is the most important hallmark of Hunter criteria, and this neuromuscular characteristic is closely related to serotonin syndrome. Although other adverse drug reactions may initially be mistaken for 5-HT toxicity, careful examination of specific neurological features, such as clonus, hyperreflexia, and tension, makes it possible to differentiate it from other conditions (Table 3). The most common confounding antipsychotic syndrome is NMS, due to the similarity of symptoms between them. Also, some patients with mental disorders take antipsychotic and antidepressant drugs at the same time to treat psychiatric disorders with depression, making it difficult to distinguish between them.

**Table 3:** Typical clinical presentation according to the Hunter criteria.

Category	Signs and symptoms
Neuromuscular	Clonus (inducible, spontaneous or ocular)
	Tremor
	Hyperreflexia
	Hypertonicity
Autonomic	Diaphoresis
	Maximum temperature > 38°C
Mental state	Agitation

We went back to the clinical course of this patient. Along with rigidity, NMS is characterized by bradyreflexia, whereas serotonin syndrome mainly manifests as neuromuscular symptoms such as myoclonus, ocular flutter, and hyperreflexia (14), which were observed in our patient. The increased muscle tone in our patient was mainly in the lower limbs rather than in all muscle groups, which differs from NMS (15). No fever was observed in our patients. In fact, almost 90% of NMS patients are characterized by a temperature above 38°C; therefore, the absence of fever in our patient was also helpful in excluding a diagnosis of NMS. Indeed, fever is not a typical symptom of serotonin syndrome (16). The patient showed significant agitation, which was different from the stupor caused by NMS (1). In addition, the patient’s symptoms continued after clozapine withdrawal, which was inconsistent with NMS.

The core symptoms of NMS are bradyreflexia, lead pipe rigidity in all muscle groups, coma, and hyperthermia, in contrast to the hyperreflexia and clonus seen in serotonin syndrome. Some of the symptoms, such as agitation, refusal to eat, nervous expression, tachypnea, CK elevation, and diaphoresis, are common to both NMS and serotonin syndrome (17). Lower limb tremors, myoclonus, and hyperreflexia are unique to serotonin syndrome. Since our patient had been taking clozapine, while the dose of escitalopram was rather low (5 mg/d), it is easy to mix up these two diagnoses. Unlike NMS, serotonin syndrome should not be considered an extremely rare idiosyncratic reaction to drugs but rather a form of 5-HT toxicity based on elevated concentrations that can occur in patients of any age (2). Considering the above, it was less likely to diagnose NMS in our patient. Thus, overall, the occurrence of hyperreflexia, increased lower extremity tension, myoclonus, agitation, ocular flutter, and high escitalopram concentration in our patient supported the diagnosis of significant serotonin syndrome.

Given that our patient continued to have certain symptoms after discontinuing clozapine and CK increased after discontinuing escitalopram, we also need to be aware of clozapine discontinuation withdrawal symptoms, which are similar to those of serotonin syndrome and include clozapine’s cholinergic withdrawal symptoms and serotonergic withdrawal symptoms (Table 4).

**Table 4:** Differences in withdrawal symptoms between serotonin syndrome, neuroleptic malignant syndrome (NMS), and clozapine discontinuation withdrawal symptoms.

	Classification	Symptoms	Core symptoms
Adverse drug reaction	Serotonin syndrome	The classic triad of clinical features is altered mental status (with anxiety, agitation, and confusion), autonomic nervous system hyperactivity (with diaphoresis, tachycardia, hyperthermia, hypertension, vomiting, and diarrhea), and neuromuscular hyperactivity (muscle rigidity, hyperkinesis that includes myoclonus and tremor, hyperreflexia, and bilateral Babinski sign).	Hyperreflexia and clonus
Idiosyncratic reaction	Neuroleptic malignant syndrome	Agitated delirium, confusion, lead pipe rigidity, cogwheel tremor, hyperthermia with body temperature greater than 40°C, profuse diaphoresis, tachycardia, hypertension, and tachypnea	Bradyreflexia, lead pipe rigidity in all muscle groups, coma, and hyperthermia
Withdrawal symptoms from clozapine discontinuation	Serotonergic symptoms	Agitation, diaphoresis, clonus, and hyperreflexia	Hyperreflexia and clonus
	Cholinergic symptoms (cholinergic rebound)	Nausea, vomiting, confusion, insomnia, and dystonia	Dystonia
	Withdrawal-associated psychosis	Delusions, hallucinations, and thought disorder (TD), including formal thought disorder (FTD) and content thought disorder	Delusions, hallucinations
	Catatonia	Stupor, posturing, and echo phenomena	Stupor, posturing, and echo phenomena

Clozapine’s cholinergic withdrawal symptoms, also known as “cholinergic rebound,” are characterized by a series of mental and physical clinical features that include nausea, vomiting, confusion, insomnia, and dystonia (18). Symptoms usually appear within a few days and last for weeks or longer. This is thought to be caused by overactivity of the cholinergic system. Although some symptoms, such as insomnia and dystonia in cholinergic rebound, partly overlap with our patient’s symptoms, the timing was not consistent with clozapine withdrawal. Before clozapine was discontinued, our patient had insomnia. In addition, cholinergic rebound does not appear as spontaneous clonus, whereas it was the core symptom of the serotonin syndrome that appeared in our patient.

Serotonergic withdrawal symptoms are very similar to those of serotonin syndrome. The only difference is the medication. Serotonergic withdrawal symptoms are usually associated with clozapine withdrawal, which is related to its 5-HT_2A_ antagonist effect. Long-term use of clozapine can lead to upregulation of serotonin receptors, and sudden withdrawal produces a response similar to serotonin syndrome (19). Theoretically, any drug with a 5-HT_2A_ antagonist effect will produce serotonin withdrawal symptoms. However, the patient’s symptoms had appeared before clozapine withdrawal. They had manifested as a more central serotonin syndrome. Therefore, this stage can rule out the possibility of serotonergic symptoms caused by clozapine withdrawal. After clozapine withdrawal, the patient still presented with the previous symptoms without showing new ones. Crucially, due to the lack of antipsychotic treatment during serotonin syndrome, the psychiatric symptoms that appeared in our patient on April 18, 2023, manifested as yelling; therefore, clozapine 50 mg was re-used for treatment. It should be noted that our patient’s psychiatric symptoms were partly controlled after treatment, while the serotonin syndrome did not improve. If the patient’s current serotonergic symptoms were caused by clozapine withdrawal, the symptoms would have disappeared after clozapine reintroduction. On the contrary, the patient’s symptoms gradually subsided after discontinuing escitalopram, ruling out the possibility that the serotonergic symptoms were caused by clozapine withdrawal. In fact, her symptoms had little to do with clozapine. Symptoms appeared when clozapine was still being taken at a dose of 275 mg; symptoms did not subside after clozapine withdrawal; symptoms did not worsen or subside when clozapine was re-introduced. This helped us exclude the diagnosis of serotonergic withdrawal symptoms in our patient after discontinuing clozapine.

In fact, there were two extreme situations in the patient that we also had to take into account, namely, the possibility of gradual regression of NMS after clozapine withdrawal and the regression of serotonergic symptoms after clozapine withdrawal. Before the symptoms appeared, our patient did not discontinue any medication, not even clozapine. After she did, the symptoms did not subside. Therefore, the possibility that the symptoms were caused by clozapine discontinuation was excluded.

**Figure 3:**
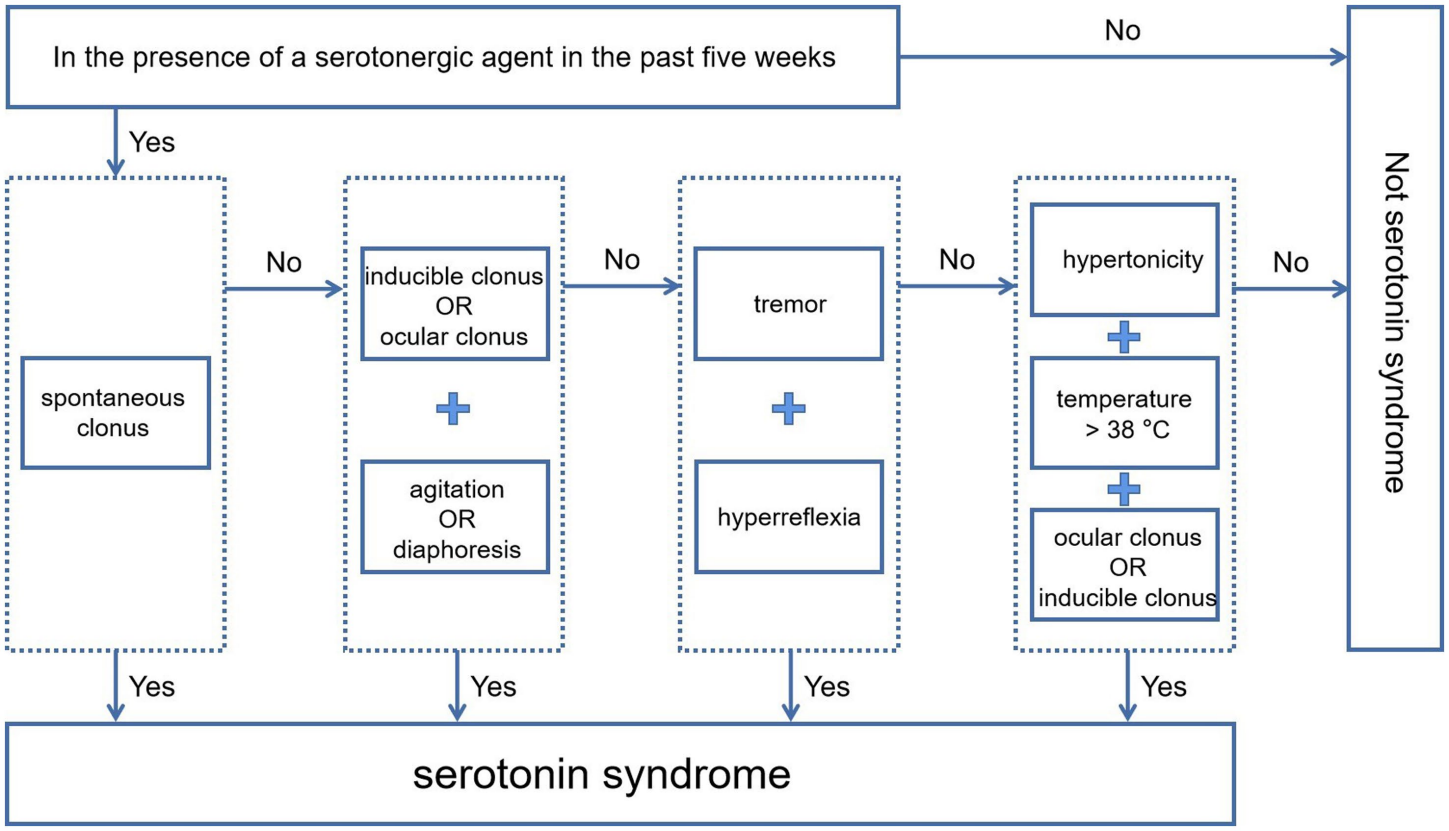
Decision rules of the Hunter serotonin toxicity criteria.

Although serotonin syndrome has many similarities to NMS, its diagnosis based on Hunter criteria is not difficult, due to some core symptoms. It is important for clinicians to be aware that patients’ current complex symptoms may be related to serotonin syndrome. Once such a connection is established, serotonin syndrome is not difficult to diagnose according to the decision rules of the Hunter Serotonin Toxicity criteria (Figure 3).

Before evaluation based on decision rules, clinicians should have a full understanding of the use of prescription drugs and overdoses, illicit substances, and dietary supplements, which is critical to determining whether Hunter criteria are applicable and whether all of these medications are related to the development of serotonin syndrome. The use of 5-HT drugs or interactions that produce 5-HT tryptamine activity is the basis of the diagnosis. Second, a detailed physical examination should be carried out. The physical examination should include a focused assessment of deep tendon reflexes, clonus, and muscle rigidity, in addition to an assessment of pupil size and reactivity, dryness of the oral mucosa, intensity of bowel sounds, skin color, and sweating. The neuromuscular features of clonus and hyperreflexia are highly diagnostic of serotonin syndrome, and their occurrence in the setting of serotonergic drug use establishes the diagnosis. Clinicians should be aware that muscle rigidity may overwhelm other neuromuscular findings and mask the diagnosis. Finally, screening for serotonin syndrome should be carried out strictly in accordance with Hunter criteria.

Cases of serotonin syndrome requiring hospitalization are easy to diagnose because severe symptoms (such as bilateral, symmetric clonus in the legs more than in the arms) are not common in other cases. The combination of non-specific autonomic manifestations, a series of possible signs and symptoms, and the lack of definitive laboratory tests makes it less likely to diagnose mild cases, although such cases are also less likely to be fatal. In addition, the risk of serotonin syndrome caused by a single therapeutic dose is low (20). The emergence of serotonin syndrome is often a combination of several drugs.

Since serotonin syndrome is a drug-induced condition, an accurate drug history is necessary for diagnosis. A wide variety of drug types and combinations can cause serotonin syndrome, and the final common pathway is thought to involve a net increase in serotoninergic neurotransmission. In addition to SSRI excess, drugs that inhibit 5-HT metabolism, increase 5-HT synthesis, increase 5-HT release, and promote 5-HT1 receptor activation can cause serotonin syndrome. There are many drug combinations that can cause serotonin syndrome. 5-HT toxicity most commonly occurs when two or more drugs that increase 5-HT are used simultaneously, especially if they increase 5-HT in different ways (21). It is worth noting that the combination of SSRI and monoamine oxidase inhibitor (MAOI) can significantly increase the risk of serotonin syndrome. SSRIs inhibit the reuptake of 5-HT and increase the level of 5-HT in the synaptic cleft. MAOIs can reduce the degradation of monoamine neurotransmitters, such as 5-HT, and significantly increase its level. Therefore, the combination of these two drugs should be strictly prohibited. The sequential use of these two drugs should also maintain a 14 day interval.

To date, there is little agreement on the effect of the serotonin concentration on serotonin syndrome. Some studies suggest that an increase in serotonin levels is likely to lead to serotonin syndrome (12). Therefore, the determination of serum 5-HT levels can be used as the basis for the diagnosis of serotonin syndrome. Other researchers believe that there is no laboratory test to confirm the diagnosis of serotonin syndrome, and the level of 5-HT concentration has no practical significance for the diagnosis (22), because the local concentration of nerve endings is the cause of the physiological effects of serotonin syndrome (23). Although the role of 5-HT concentration in serotonin syndrome is still controversial, most studies generally believe that high doses of SSRI drugs or drug behaviors that can produce high serotonin effects can lead to serotonin syndrome (2, 24). The concentration of escitalopram in our patient reached a staggering 210.9 ng/mL, exceeding the warning level, which significantly supported the diagnosis of serotonin syndrome.

Aggregation of 5-HT caused by high concentrations of SSRIs leads to serotonin syndrome, which was confirmed by the blood concentration in our patient, but how did she reach such a high concentration with a relatively low dose of escitalopram? Drug interactions are a factor that cannot be ignored. Escitalopram is a well-known SSRI that is mainly metabolized by CYP2C19, a highly polymorphic enzyme known to cause individual differences in pharmacokinetics (25). Clopidogrel is a thiophene-pyridine prodrug that also needs to be metabolized by CYP2C19 to form active thiol derivatives that selectively and irreversibly inhibit P2Y_12_ receptors (26). The serotonin syndrome here seemed to occur due to an interaction between these two drugs. To date, only escitalopram-increasing effects of clopidogrel through pharmacodynamic synergism have been reported (27), and there are no such reports of clopidogrel-increasing effects on the pharmacodynamics and pharmacokinetics of escitalopram. In light of this, we examined the CYP2C19 polymorphism and surprisingly discovered that it was typical of poor metabolizers (PMs), as evidenced by the presence of two nonfunctional alleles (CYP2C19 *2/*3). The metabolism of both escitalopram and clopidogrel was slowed down. That means, at first, the serum level of escitalopram slowly increased or balanced as a result of the comprehensive effect of poor metabolism of CYP2C19, continuous exogenous intake, and metabolism. However, clopidogrel, which is metabolized by the same enzyme, was also introduced at the same time, which seriously slowed down the metabolism of escitalopram. As a result, one month later, the level of escitalopram steadily increased to a dangerous level, resulting in serotonin syndrome. It should be noted that our patient was taking clozapine, an atypical antipsychotic drug with partial 5-HT_2A_ antagonism, which can also delay serotonin syndrome (28). The withdrawal of clozapine may, instead, partially aggravate the symptoms of serotonin syndrome. Therefore, overall, explaining the reason how serotonin syndrome caused by low-dose of escitalopram and why the syndrome developed so slowly.

We do not believe that our patient developed serotonin syndrome using such a low dose of escitalopram alone. The patient’s platelet control was acceptable during the combination of clopidogrel and escitalopram, whereas gastric bleeding symptoms occurred after the discontinuation of escitalopram, indicating a significant increase in clopidogrel active metabolites after CYP2C19 metabolism. This may be the initial combination of overloaded drug metabolism causing CYP2C19 to “run at full load” due to its PMs. Although escitalopram can affect platelet activation and increase the effect of clopidogrel, clopidogrel activation is reduced due to the PMs of CYP2C19, and patients have no risk of bleeding. When escitalopram was removed, CYP2C19 remained operating at full load and then converted more clopidogrel to an active metabolite, thus resulting in gastric bleeding. This further indicates that there is a co-occupancy of CYP2C19 in the combination of escitalopram and clopidogrel, causing a decrease in the metabolism of both drugs. To put it another way, escitalopram, when taken alone at a dose of 5 mg/d in our patient, does not lead to accumulation and is metabolized by CYP2C19, similarly to clopidogrel. It is precisely because of the combination of the two drugs, which are both metabolized by CYP2C19, and the patient’s low enzyme activity leads to serotonin syndrome.

Other drugs that may affect the metabolism of CYP2C19 may also affect the metabolism of escitalopram, e.g., enzyme inhibitors and enzyme inducers. Inhibitors can hinder the metabolism of escitalopram, increase its concentration in the body, and cause drug overdose poisoning. Inducers may accelerate the metabolism of escitalopram and reduce its efficacy. CYP2C19 enzyme inhibitors and inducers are shown in Table 5.

**Table 5:** CYP2C19 enzyme inhibitors and inducers.

Classification	Inhibitor	Inducer
	Esomeprazole, Omeprazole, Fluconazole, Voriconazole, Chloramphenicol, Artemisinin, Isoniazid, Fluoxetine Hydrochloride, Indomethacin, Sodium Valproate, Oxcarbazepine, Fluvastatin, Lovastatin, Nicardipine, Amiodarone, Zafirlukast, and Oral contraceptives, etc.	Rifampicin, ritonavir, dexamethasone, Ginkgo preparations, and St. John’s wort

To our knowledge, there have been no reports of serotonin syndrome caused by low-dose escitalopram in combination with clopidogrel. Only one case has been reported that was caused by low-dose citalopram. The report described a 40-year-old male patient who developed serotonin syndrome within 3 h of taking citalopram 30 mg (29). The effective dose of escitalopram in this case was relatively high, in contrast to our patient. Since it is generally associated with high doses of serotonergic drugs, the serotonin syndrome in our patient is quite rare. Meanwhile, cases of serotonin syndrome related to poor metabolizers of CYP2D6 have also been reported (30, 31).

## Conclusion

In older adults on low-dose antidepressants, drug interactions, and low metabolic enzyme activity can lead to serotonin syndrome, which is likely to be ignored by doctors in the majority of cases. Additionally, it is easy to overlook mixed symptoms when dealing with serotonin syndrome, and unintentionally increasing medication dosages or adding medications with high levels of 5-HT could result in serious clinical events.

Although the combination treatments of clopidogrel and escitalopram did not cause serotonin syndrome in most patients, it is still necessary to be alert for drug accumulation in patients with poor metabolism of CYP2C19, which may still be fatal in some special cases. Early detection, TDM and genetic testing, active intervention, and treatment are essential for the further management of serotonin syndrome.

## Data availability statement

The original contributions presented in the study are included in the article/supplementary material, further inquiries can be directed to the corresponding authors.

## Ethics statement

Written informed consent was obtained from the individual(s) for the publication of any potentially identifiable images or data included in this article. Written informed consent was obtained from the patient and her relatives, and the information was de-identified to protect anonymity.

## Author contributions

JW: Writing – original draft. JY: Writing – original draft. KQ: Validation, Writing – review & editing. JJY: Validation, Writing – review & editing. CZ: Validation, Writing – review & editing. XL: Validation, Writing – review & editing.
